# Regulation of asymmetric positioning of nuclei by Wnt and Src signaling and its roles in POP-1/TCF nuclear asymmetry in *Caenorhabditis elegans*

**DOI:** 10.1111/j.1365-2443.2010.01388.x

**Published:** 2010-04

**Authors:** Kenji Sugioka, Hitoshi Sawa

**Affiliations:** 1 Laboratory for Cell Fate Decision, RIKEN, Center for Developmental BiologyKobe 650-0047, Japan; 2 Department of Biology, Graduate School of Science, Kobe UniversityKobe 657-8501, Japan

## Abstract

In various polarized cells, positions of nuclei are often off-center. However, extrinsic signals regulating nuclear off-centering and its biologic roles remain to be elucidated. In *Caenorhabditis elegans*, polarity of the EMS cell undergoing asymmetric division is regulated by the MOM-2/Wnt and MES-1 signals from its posterior neighbor P2 cell. We show that after divisions of different cells including EMS, the nuclei of the posterior but not anterior daughter cells are anchored to the posterior cell cortex via centrosomes. We also show that this nuclear anchoring is regulated by components of the Wnt pathway and SRC-1 that functions in MES-1 signaling. To understand the biologic roles of nuclear anchoring, we analyzed its effects on asymmetric nuclear localization of POP-1/TCF that is also regulated by Wnt and Src signaling. We found that in *mom-2* mutants where the nuclear anchoring and POP-1 asymmetry is partially inhibited, the proximity of the nucleus to the cell cortex correlated with POP-1 asymmetry. Furthermore, in mutants of *mom-2*, the defect in the anchoring is clearly correlated with that of asymmetric fate determination. These results suggest that the asymmetric nuclear anchoring functions in asymmetric division by enhancing POP-1 asymmetry.

## Introduction

Asymmetric division is a fundamental way to produce cellular diversity. For cells to divide asymmetrically, they must be polarized by extrinsic and/or intrinsic cues. Then, the cell polarity enforces asymmetric localization of cell fate determinants. Asymmetric segregation of the localized determinants is achieved by proper orientation of the spindle along the axis of the polarity (Gönczy 2008; Siller & Doe 2009). In *Caenorhabditis elegans (C. elegans)*, asymmetric division is one of the important processes for establishing the body plan. Asymmetries of many divisions along the anteroposterior axis are regulated by a Wnt pathway called Wnt/β-catenin asymmetry pathway (Mizumoto & Sawa 2007b). For example, the embryonic EMS cell is polarized by the MOM-2/Wnt signal from its posterior neighbor, the P2 cell. This triggers the Wnt effector DSH-2/Dishevelled and WRM-1/β-catenin to be localized to the posterior and anterior cell cortex, respectively (Walston *et al.* 2004), (Nakamura *et al.* 2005). At telophase, when the nuclear membrane is reformed, WRM-1 starts to localize preferentially to the posterior nucleus (Nakamura *et al.* 2005) where it promotes nuclear export of POP-1/TCF (Lo *et al.* 2004; Mizumoto & Sawa 2007b), creating reciprocal asymmetry of nuclear WRM-1 and POP-1. The asymmetry of POP-1 localization (POP-1 asymmetry) results in its distinct transcription activities and asymmetric cell fate decision in the daughter cells (MS and E) (Phillips & Kimble 2009). It was also reported that SRC-1/Src tyrosine kinase cooperates with Wnt signaling in the regulation of POP-1 asymmetry and the orientation of the mitotic spindle during the EMS division (Thorpe *et al.* 1997; Schlesinger *et al.* 1999; Bei *et al.* 2002). However, how Wnt and Src signaling control asymmetric WRM-1/β-catenin and POP-1/TCF nuclear localization remains to be elucidated.

In this study, we found that during telophase of the EMS division, the posterior but not anterior centrosomes moved toward the cell cortex and were attached to it just after the division to anchor the E nucleus to the cell periphery (nuclear anchoring) in a manner dependent on Wnt and Src signaling. In *mom-2/wnt* mutants, in which the asymmetric nuclear anchoring was partially disrupted, POP-1 asymmetry was more strongly affected in embryos with E nuclei located far from the cortex than compared to those with E nuclei located close to the cortex. Therefore, our results suggest a novel role for the positioning of nuclei in the regulation of asymmetric division.

## Results

### The posterior nucleus is anchored to the cell cortex by centrosomes just after the EMS division

**Figure 1:**
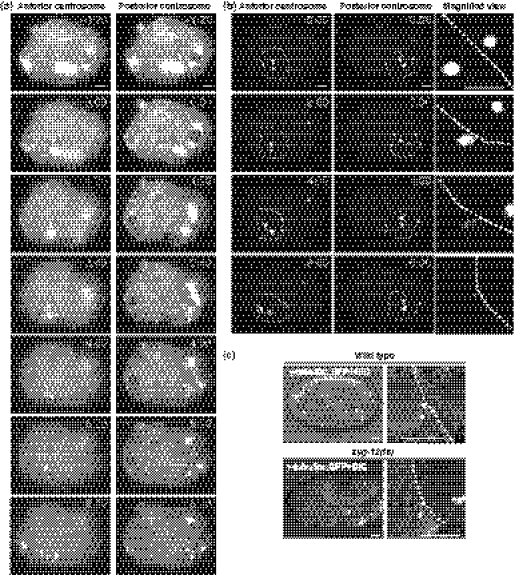
The nucleus of posterior daughter cell is anchored to the cell cortex by centrosomes after an asymmetric division. (a, b) Time-lapse images of the same embryos expressing GFP::β-tubulin (a) or GFP::γ-tubulin (b) in the focal planes in which the center of anterior or posterior centrosomes was clearly observed as indicated in the top of the panels. The numbers on the left indicate the amount of time (minutes : seconds) after furrowing onset. Asterisks indicate the center of the anterior or posterior nuclei. Arrowheads indicate the positions of centrosomes. Both centrosomes collapsed and duplicated around 4:00. Some of the duplicated centrosomes were out of focus in the images after 5:20. In (b), the boundaries of the MS and E cells are outlined by dotted lines in the left and center panels, respectively. The right panels show the magnified images of the middle panels around the posterior side of the E cell with its boundary to the P2 cell outlined by dotted lines. (c) Merged differential interference contrast (DIC) and GFP images of wild-type or *zyg-12(ts)* embryos expressing GFP::γ-tubulin at 6 min after furrowing onset. The right panels show the magnified images of the left panels around the posterior side of the E cell with their boundaries to the P2 outlined by dotted lines. Arrowheads and asterisks indicate the positions of centrosomes and center of the nuclei in the E cell, respectively. Bars, 5 μm.

To analyze microtubule organization during and after the asymmetric EMS division, we performed the 4D live imaging of green fluorescent protein (GFP)::β-tubulin and GFP::γ-tubulin during the division (Fig. 1). When the cell entered mitosis, the axis of centrosomes rotated from left–right to anteroposterior orientations, as reported previously (Hyman & White 1987). At late telophase, we found that the posterior but not the anterior centrosomes elongated along the anteroposterior axis with its posterior end reaching to the posterior cortex (Fig. 1a; 2:40, Fig. 1b,c; 2:00). Within 1 min after centrosome duplication that occurs soon after the completion of the division, at least one of the duplicated centrosomes in the posterior daughter E cell were always attached to the cell boundary between the E and P2 cells (P2/E boundary) and were sandwiched between the E nucleus and the cell cortex (Fig. 1 and Table 1). The centrosomes and the nucleus remained attached to the cortex until approximately 10 min after the division. In contrast, such cortical attachment was rarely (3%) observed for the centrosomes in the anterior MS daughter (Fig. 1 and Table 1). The peripheral positioning of nuclei but not centrosomes was reported previously in some embryonic cells including the E cell (Schierenberg 1987; Goldstein 1995). To know whether the centrosome–nucleus interaction is required for the peripheral positioning of the E cell nucleus, we observed embryos with a mutation in the *zyg-12* gene that encodes a protein with a KASH domain and is required for the attachments of centrosomes to nuclear membrane (Malone *et al.* 2003). When we shifted *zyg-12* temperature-sensitive mutants to the restrictive temperature just before the EMS division, the posterior nucleus appeared to be attached to the elongated centrosome at late telophase, but dissociated from the centrosome before its attachment to the cortex (100%*n* = 14, Fig. 1c). In addition, a temperature up-shift of *zyg-12(ts)* mutants after the establishment of the centrosome–cortex attachment caused detachment of the E nucleus from the cell cortex (data not shown). These results suggest that the E cell nucleus is continuously anchored to the P2/E boundary via the centrosome–nucleus interaction (hereafter called the nuclear anchoring).

**Table 1:** Nuclear anchoring in Wnt and Src knockdown embryos

Cell	Genotype	% Cen0	% Cen1	% Cen2	*n*	*P*-value
E	WT	0	29	71	41	NA
E	*mom-2(RNAi)*	17	44	39	18	0.007
E	*src-1(RNAi)*	80	20	0	15	<0.0001
E	*mom-5(RNAi)*	50	40	10	10	<0.0001
E	*dsh-2;mig-5(RNAi)*	70	20	10	10	<0.0001
E	*gsk-3(RNAi)*	100	0	0	8	<0.0001
E	*mig-14(or78)*	60	31	9	45	<0.0001
E	*wrm-1(RNAi)*	0	33	67	9	1.0
E	*apr-1(RNAi)*	0	17	83	6	1.0
E	*pop-1(RNAi)*	0	62	38	8	0.11
ABalp	WT	0	49	51	35	NA
ABalp	*mom-2(RNAi)*	31	46	23	13	0.004
ABalp	*src-1(RNAi)*	100	0	0	11	<0.0001
ABprp	WT	0	21	79	14	NA
ABprp	*mom-2(RNAi)*	56	11	33	9	0.005
ABprp	*src-1(RNAi)*	100	0	0	7	<0.0001

**Figure 2:**
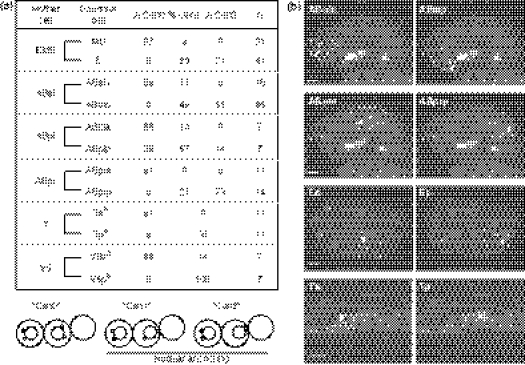
The nuclear anchoring in various cells. (a) The positions of nuclei and centrosomes in indicated embryonic daughter cells were observed at 320 s after furrowing onset (approximately 2.5 min after the completion of the divisions) of their mother cells (See also Fig. S1 in Supporting Information). For postembryonic T and V5 cells, nuclei were observed at late telophase or just after the division. In each daughter cell pair, the upper and lower rows in the table indicate anterior and posterior daughter cells, respectively. Cen0, Cen1 and Cen2 indicate that zero, one and two centrosomes, respectively, were observed to be attached to the cortex as schematically described at the bottom of the table. In all cases, centrosomes were attached to nuclei. Nuclei were anchored to the cortex in Cen1 and Cen2. (b) Left and right are images of the same embryos in the focal planes with focuses on the anterior and posterior daughter cells, respectively. Cell boundaries are outlined by dotted lines. Asterisks and arrowheads indicate the center of the nuclei and the positions of centrosomes, respectively. Bars, 5 μm.

Cen0, Cen1 and Cen2 phenotypes are described in Fig. 2a. *P*-values were calculated by Fisher’s exact test.

### Attachments of nuclei to the cell cortex in various posterior daughter cells

We also analyzed positions of nuclei and centrosomes in some other embryonic and postembryonic cells shown in Fig. 2a just after their birth to find that they also attached to the posterior cell cortex with varying degrees in most posterior but not anterior daughter cells we examined (Fig. 2) except for the daughters of ABar that divides nearly along the left–right axis (Thorpe *et al.* 1997). In the posterior sister cells, whenever we observed cortical attachments of nuclei, their centrosomes were observed between the nuclei and the cell cortex (data not shown). This strong correlation suggests that the nuclei are anchored to the cortex by centrosomes also in these cells.

### Wnt and Src signaling regulate the nuclear anchoring via centrosomes

**Figure 3:**
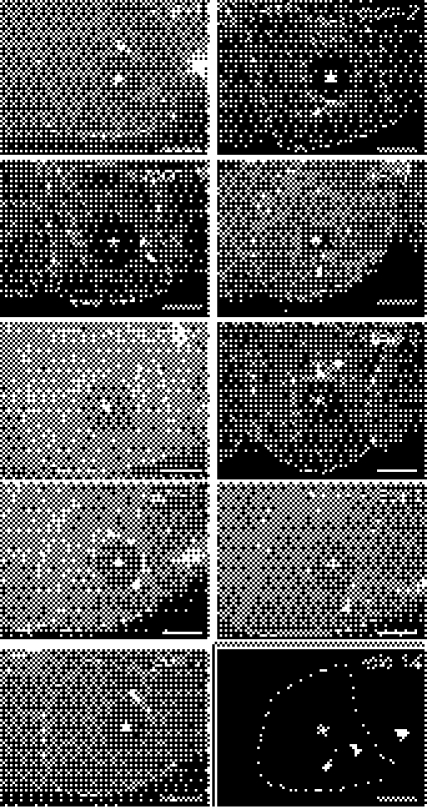
Wnt and Src signaling regulate the nuclear anchoring. Images of GFP::β-tubulin expressing embryos at 5:20 after furrowing onset after RNA interference inhibition of genes indicated on each panel, except for the image of *mig-14* which is the localization of GFP::γ-tubulin in *mig-14(or78)* mutants. Bars, 5 μm.

**Figure 4:**
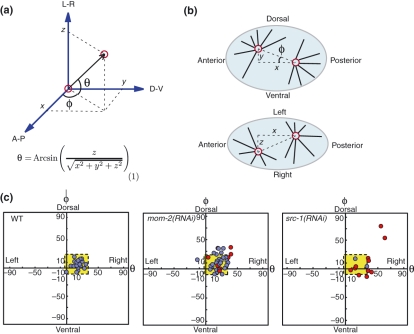
The nuclear anchoring and spindle orientation are independently regulated by Wnt and Src signaling. (a, b) Calculation of mitotic spindle orientation, using three-dimensional coordinate geometry. When embryos are mounted on glass slides, they are usually mounted so that their *L*–*R* axis corresponds to the *z*-axis. Spindle orientation was determined using Cartesian coordinate grid along the anteroposterior, dorsoventral and left–right axes by measuring *x*, *y*, *z* at furrowing onset, with the posterior centrosome as the origin. The orientation relative to the anteroposterior axis (φ) was also measured, and orientation relative to the focal plane (θ) was calculated from x, y and z using equation in (a). (c) Plots of φ and θ representing spindle orientation in wild-type, *mom-2(RNAi)* or *src-1(RNAi)* embryos. Blue and red circles indicate normal and defective nuclear anchoring, respectively, at 320 s from furrowing onset. The normal range of angles to which wild-type samples is confined is indicated by the yellow box in each panel.

It was reported previously that the proximity of the E cell nucleus to the P2/E boundary requires the P2 cell which is on the posterior side of E (Schierenberg 1987; Goldstein 1995), suggesting that signals from the P2 cell regulate the nuclear anchoring. It is known that the P2 cell sends at least two signals to EMS to regulate its asymmetric division (Thorpe *et al.* 1997; Bei *et al.* 2002). They are mediated by MOM-2/Wnt and a transmembrane protein MES-1 that functions through the Wnt/β-catenin asymmetry pathway and SRC-1 tyrosine kinase, respectively. Therefore, we analyzed the functions of these signaling pathways in the anchoring of the E nucleus to the cortex and found the attachment defect in mutants or RNA interference (RNAi) embryos of SRC-1 or of some Wnt signaling components (MOM-2, MOM-5/Frizzled, MIG-14/Wntless, DSH-2;MIG-5/Dsh, GSK-3/Gsk3β) [MIG-14 and Wntless are required for the secretion of Wnts (Coudreuse & Korswagen 2007)] (Fig. 3 and Table 1). The defect was not observed by RNAi of more downstream components of Wnt signaling (APR-1/APC, WRM-1, and POP-1). Similar effects of *mom-2* and *src-1* were also observed for the nuclear anchoring in ABalp and ABprp (Table 1). These results suggest that both Wnt and Src signaling regulate the nuclear anchoring to the cell cortex via centrosome. However, because all these components required for the anchoring are also known to regulate spindle orientation in EMS (Thorpe *et al.* 1997; Schlesinger *et al.* 1999; Bei *et al.* 2002), the defect in centrosome–cortex attachment in these mutants might be the secondary consequences of the spindle orientation defect. To exclude this possibility, we analyzed correlations between these phenotypes by observing spindle orientation at the onset of cytokinesis and then, 320 s later, the centrosome–cortex attachment in live *mom-2(RNAi)* and *src-1(RNAi)* embryos. To quantify spindle orientation in intact embryos in three dimensions, as shown in Fig. 4a,b, we measured the angle (φ) of the spindle relative to the anteroposterior axis and calculated another angle (θ) of the spindle relative to the focal plane from distances (*x*, *y* and *z*) between centrosomes along the three body axes. In control wild-type embryos, EMS spindle orientation plots were concentrated in a small area (i.e. normal range, indicated by the yellow box in Fig. 4c) with limited variability and was slightly tilted toward the right. In *mom-2/*Wnt*(RNAi)* or *src-1(RNAi)* embryos, spindle orientation was often plotted outside of the normal range, consistent with the orientation defects reported previously (Schlesinger *et al.* 1999; Bei *et al.* 2002). In four of seven *mom-2* and three of 11 *src-1* embryos among those that were defective in the anchoring of E nuclei (red circles in Fig. 4c), spindle orientation was plotted in the normal range, indicating that the anchoring defect was not caused by abnormal spindle orientation at least in these embryos, although the anchoring defect in the remaining embryos (red circles outside of the yellow area) might be caused or influenced by the orientation defect. These results strongly suggest that the anchoring of E nucleus is regulated by Wnt and Src signaling independently of their roles in spindle orientation.

### The roles of the nuclear anchoring in asymmetric division

**Figure 5:**
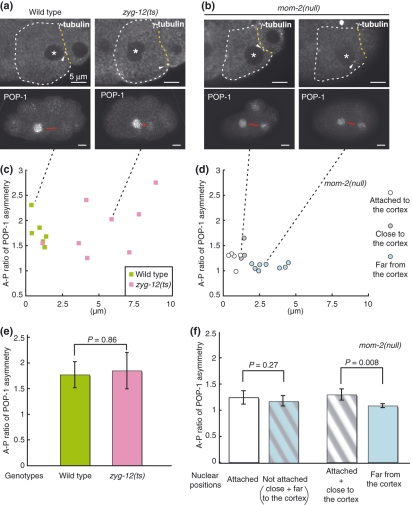
The nuclear anchoring potentiates POP-1 asymmetry. (a, b) Examples of wild-type, *zyg-12(ts)* and *mom-2(or309)* embryos at 7:00 relative to furrowing onset stained for POP-1 and γ-tubulin. Red bars indicate the positions of sister nuclei. Cell boundaries of the E cell are outlined with white dotted lines except for the P3/E boundary with yellow dotted lines. Black dotted lines below the images indicate samples in (c) and (d) corresponding to the embryos in (a) and (b), respectively. (c, d) Ratios of POP-1 asymmetry between the MS and E nuclei (A–P ratio) in wild-type (green squares) and *zyg-12(ts)* embryos (pink squares) in (c), and *mom-2(or309)* embryos in (d) were plotted over the distances between the posterior edge of nuclei and the P3/E boundary. In (d), white, gray and blue circles indicate that the nuclei were attached to the P3/E boundary, close to but not anchored to it and far from it, respectively, judged by visual observation by DIC microscopy at 6 min from furrowing onset before fixation. The distances shown in the panel were measured after staining. The staining procedure might cause slight shifts of nuclear positions, so that the distances were variable among embryos with the normal nuclear anchoring (white circles), although the distances were still shorter compared to embryos without the nuclear anchoring. (e, f) Average A–P ratios of POP-1 asymmetry in wild type, *zyg-12(ts)*, *mom-2(or309)* mutants calculated from the data in (c) and (d). Error bars indicate the standard error of mean (SEM) at 95% confidence. *P*-values were calculated by Mann–Whitney *U*-test. Significant differences were not detected in comparisons between wild type and *zyg-12* mutants (e), and those between *mom-2* mutants with (white circle in d) and without (gray + blue circles in d) nuclear attachment to the P3/E boundary (left two bars in f). *mom-2* mutants with their E nuclei attached or close to the cortex (white + gray circles in d) showed significantly higher POP-1 asymmetry than those (blue circles in d) with their nuclei far from the cortex (right two bars in f). White bars, 5 μm.

The roles of the peripheral positioning of nucleus have not been analyzed previously in *C. elegans*. Because the nuclear anchoring in the E cell results in asymmetric nuclear positioning between the MS and E sister cells, we thought that the nuclear anchoring might influence other asymmetries between the sister cells, i.e., POP-1 asymmetry and/or the difference of cell fates. We first examined correlations between the nuclear anchoring and POP-1 asymmetry using the temperature-sensitive mutants of *zyg-12*/KASH. In these experiments, in live embryos, we visually judged the attachment and the proximity of E nuclei to the P2/E boundary at approximately 3 min after the completion of the division and immediately fixed and stained them for γ-tubulin and POP-1/TCF (Fig. S2). The proximity was confirmed after staining by measuring the distance between nuclei and the posterior cortex. In *zyg-12* mutants, although the nuclear anchoring completely failed, POP-1 asymmetry was similar to the wild-type level (Fig. 5a,c,e), indicating that the nuclear anchoring is not essential for POP-1 asymmetry that is mainly regulated by Wnt signaling. Next, we examined the possible functions of the nuclear anchoring in the *mom-2*/Wnt*(null)* sensitized background, because *mom-2(null)* mutants were not completely defective in asymmetric cell fate determination (83% defective *n* = 30) and POP-1 asymmetry (Fig. 5d). Firstly, we compared the POP-1 asymmetry between *mom-2* mutants with and without the cortical attachment of nuclei in the E cell (white versus gray + blue circles in Fig. 5d and the left two bars in Fig. 5f), but we could not detect significant effects even in the *mom-2* background. We noticed, however, that the animals whose E nuclei are close to the cell cortex (white + gray circles in Fig. 5d) showed significantly higher POP-1 asymmetry compared to those with nuclear positions far from the cell cortex (blue circles in Fig. 5d), as shown in Fig. 5b and the right two bars in Fig. 5f. These results suggest that the proximity between the nucleus and the cell cortex is important to potentiate POP-1 asymmetry in the background sensitized by the *mom-2(null)* mutation. The nuclear attachment to the cortex by itself may not be important, but the nuclear anchoring is required to keep the nucleus in the proximity to the cortex.

We further analyzed the correlations between abnormal nuclear anchoring and cell fate defects in *src-1(RNAi)*, *mom-2(or309)* and *mig-14(or78)/*Wntless mutants. We observed the nuclear anchoring phenotype of live embryos and subsequently analyzed the asymmetry of cell fates by observing differentiation of gut produced from the E cell by its autofluorescence. As reported previously (Bei *et al.* 2002), *src-1(RNAi)*, animals show normal gut differentiation (100%*n* = 15), although 80% of them had the anchoring defect (Table 1), confirming that the nuclear anchoring is not important in the background with full *mom-2* activity. However, in *mom-2/*Wnt or *mig-14*/Wntless mutants, all the embryos with defective anchoring did not produce gut (10 of 30 and 27 of 45 total embryos in *mom-2* and *mig-14*, respectively), whereas only some embryos with normal anchoring showed gut differentiation (25%*n* = 20 and 28%*n* = 18 in *mom-2* and *mig-14*, respectively). These results indicate that asymmetric nuclear anchoring is involved in asymmetric cell fates determination at least when the Wnt signal is abrogated.

## Discussion

According to Hertwig’s rules described in Wilson’s (1925) textbook, the nucleus tends to take up a position at the center of a cell. In fission yeast, nuclear centering is achieved by the active microtubule-dependent pushing force (Toda *et al.* 1983; Umesono *et al.* 1983; Tran *et al.* 2001; Daga *et al.* 2006). In polarized cells, however, nuclear positions are often displaced from the center by nuclear migration and anchoring. For example, in migrating neuronal cells derived from mammalian forebrain, forward movement of nuclei is preceded by that of centrosomes (Metin *et al.* 2008). The nuclear migration and anchoring events in a variety of organisms involve the protein complexes containing KASH and SUN family proteins which bridge inner and outer nuclear membrane and transfer forces from the cytoplasm to the nucleus (Starr 2009). For example, in the *Drosophila* eye disc, the nuclei of photoreceptor cells are positioned near the apical surface via centrosome through the function of a KASH protein Klarsicht (Patterson *et al.* 2004). Similarly, we showed, in *C. elegans*, that the nucleus is anchored by the centrosomes to the cell cortex in a ZYG-12/KASH-dependent manner. This process in *C. elegans* has two unique features, which have not been reported in other systems. First, nuclear anchoring occurs asymmetrically only in the posterior daughter cells after asymmetric divisions. Second, the anchoring is regulated by Wnt and Src signaling at least in the embryonic EMS, ABal and ABpr divisions. Considering the observations that the nuclear anchoring was observed in many other embryonic and postembryonic divisions and that DSH-2/Dishevelled is localized to the posterior cortex where the nuclei are anchored in the T.p and V5.p cells as in the EMS cell (Walston *et al.* 2004; Mizumoto & Sawa 2007a), Wnts may regulate nuclear anchoring throughout the *C. elegans* development. In other organisms as in *C. elegans*, Wnt and/or Src signaling might regulate nuclear positioning or anchoring in polarized cells.

How is the nuclear anchoring achieved? Before the nuclear anchoring, Wnt and Src signaling is thought to regulate spindle orientation through the pulling force from the P2/EMS boundary where the G protein regulator complex (GPR-1/GPR-2/LIN-5) and DNC-1/dynactin localized in Src and Src + Wnt-dependent manners, respectively (Srinivasan *et al.* 2003; Tsou *et al.* 2003; Zhang *et al.* 2008). Therefore, it may be that similar mechanisms pull the posterior centrosome to the cortex. Interestingly, in contrast to prophase–anaphase when the spindle vigorously oscillated vertically, during the process of centrosome attachment to the cortex, spindle oscillation became milder (data not shown) and posterior centrosomes were slightly elongated toward the P2/EMS boundary (Fig. 1b; 2:00). This may suggest that nature of pulling force from the posterior cortex is different from that before anaphase. In addition, the dynactin localization at the P2/EMS boundary disappears before the telophase of the EMS division (Zhang *et al.* 2008). Therefore, the components of force generators and properties of the force may somehow differ between spindle orientation and the nuclear anchoring.

**Figure 6:**
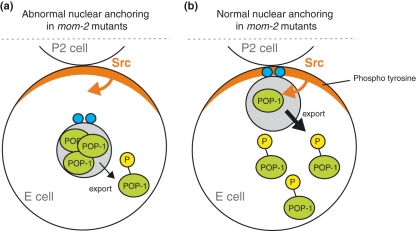
A model for the function of nuclear anchoring in the POP-1 regulation in *mom-2* mutants. (a) When the nucleus is apart from the cell cortex, the Src activities at the cell cortex cannot facilitate POP-1 phosphorylation by LIT-1 kinase required for POP-1 nuclear export (Lo *et al.* 2004), resulting in the accumulation of nuclear POP-1. (b) When the nucleus is anchored to the cell cortex, the Src activities at the cell cortex facilitate POP-1 phosphorylation, resulting in lower nuclear POP-1 level. Nuclei and centrosomes are indicated by gray and blue circles, respectively.

The biologic roles of nuclear anchoring still remain to be understood. In mammalian skeletal muscle where three to six nuclei are anchored to the postsynaptic membrane at the neuromuscular junctions (Sanes & Lichtman 2001), it was suggested that the nuclear anchoring strengthens communication between nerve and muscle, because, in the absence of KASH protein Syne-1/Syne-2, the morphology of motor neurons becomes abnormal (Zhang *et al.* 2007). In the asymmetric division of the EMS cell, although the nuclear anchoring itself is not required for POP-1 asymmetry and cell fates in *mom-2*/Wnt (*+*) animals, we showed that it has a significant influence on POP-1 asymmetry and asymmetric cell fates in the absence of MOM-2. Because the E cell nucleus attaches to the P2/E boundary, where Src-dependent tyrosine phosphorylation is enriched even in *mom-2* mutants (Bei *et al.* 2002), we expect that the Src signaling may be more efficiently transduced to create weak POP-1 asymmetry when the nucleus is anchored in *mom-2* mutants (Fig. 6). In any signaling systems, it is plausible to imagine that positioning of the nucleus near the source of the signals can facilitate transduction of the signals. Therefore, nuclear positioning may play key roles in many signaling systems including Wnt signaling in other organisms.

## Experimental procedures

### Strains and alleles

All *C. elegans* strains used in this study were cultured by standard methods (Brenner 1974). *zyg-12(or577ts)* mutants were grown at 15 °C and their embryos were shifted to 25 °C before observation. The Bristol strain N2 was used as wild type. The following integrated transgenic lines were used: GFP-γ-tubulin *[unc-119(+) pie-1 promoter::gfp::tbg-1]* (Oegema *et al.* 2001); GFP-β-tubulin *[unc-119(+) pie-1 promoter::gfp::tubulin]* (Praitis *et al.* 2001); γ-tubulin-GFP *[pRF4 + tbg-1::gfp]* (Bobinnec *et al.* 2000).

### RNA interference

For production of dsRNA, the following cDNAs were used as templates: yk40c12 (*apr-1*), yk117f2 (*src-1*), yk213d6 (*wrm-1*), yk233b4 (*gsk-3*), yk471e5 (*mom-5*), yk714f1 (*mom-2*), yk55h11 (*dsh-2*), yk216a12 (*mig-5*) and pMM414 (*pop-1*) (Maduro *et al.* 2002). Embryos were collected 24–40 h postinjection.

### Microscopy and analysis of living embryos

All the embryos were dissected from gravid hermaphrodites in egg salt buffer (Edgar 1995). For most experiments, the embryos were mounted on 5% agarose pads under coverslips and sealed with Vaseline. Embryos were observed at room temperature with a Plan-Apochromat 100× 1.4 NA oil immersion lens by using a CSU10 spinning-disc confocal system (Yokogawa Electric Corp., Tokyo, Japan) mounted on an AxioPlan2 microscope (Carl Zeiss, Inc, Oberkochen, Germany). The specimens were illuminated with a diode-pumped solid state 488-nm laser (HPU50100, 20 mW; Furukawa Electronic, Tokyo, Japan). Images were acquired with an Orca ER12-bit cooled CCD camera (Hamamatsu Photonics, Hamamatsu, Japan), and the acquisition system was controlled by IP lab software (2 × 2 binning; Scanalytics, Inc, Rockville, MD, USA). Acquired images were processed with the IP Lab software and Adobe Photoshop (Adobe Systems, San Jose, CA, USA). In the analysis of the effects of the nuclear anchoring on cell fate asymmetry, we judged nuclear attachment by differential interference contrast (DIC) microscopy at room temperature and then incubated the slides at 15 °C over night and observed the autofluorescence of gut granules.

### Immunostaining and quantification of POP-1 protein levels

For staining of γ-tubulin and POP-1, embryos at 7 min after furrowing onset (approximately 3 min after the completion of the division) of the EMS cells were freeze-cracked, and slides were fixed in −20 °C methanol for 5 min and rehydrated 2 min each through 95%, 90%, 75%, 50% and 30% methanol series diluted in phosphate buffered saline supplemented with Tween-20 (PBSTw). After washing three times with PBSTw, slides were incubated with the mouse anti-POP-1 antibody (clone P4G4, 1 : 2500; Lin *et al.* 1998) and the rabbit anti-γ-tubulin antibody (LL-17; 1 : 1000; Sigma-Aldrich, St. Louis, MO, USA) in PBSTw supplemented with 1% BSA at 4 °C over night. After washing three times, slides were incubated with the Rhodamine-X-conjugated goat anti-mouse IgG (1 : 1000; Invitrogen, Carlsbad, CA, USA) and Fluorescein-conjugated goat anti-rabbit IgG (1 : 1000; Invitrogen) for 2 h at room temperature. POP-1 asymmetry was calculated as the ratio of the average fluorescence intensities between the anterior and posterior nuclei quantified using the IP lab software.

## Supporting Information/Supplementary material

**Figure S1** Schematic views of *Caenorhabditis elegans* embryos with centrosome positions. Left side is up for embryos at the 6- and 14-cell stages (top and bottom). For the 8-cell and 12-cell stages, embryos on the left and right sides of the black dotted line are shown with their left side up and right side up, respectively. Double headed arrows and thin black lines indicate the orientation of the divisions and sister cell pairs, respectively. Centrosomes of posterior daughter cells which attached to the cell cortex and those which did not are shown as small red and gray circles, respectively.

**Figure S2** Timeline of the experimental procedures used in Fig. 5. Experimental procedures and cellular events were plotted over time. 0 min is a furrowing onset. Note that we judge the nuclear attachment before fixation and measured the distance between nuclei and cell cortex after staining.
