# Supplementary figures and images for: Regulation of asymmetric positioning of nuclei by Wnt and Src signaling and its roles in POP-1/TCF nuclear asymmetry in Caenorhabditis elegans
Source: Genes Cells. 2010 Apr;15(4):397–407. doi: 10.1111/j.1365-2443.2010.01388.x (PMC2855865; doi:10.1111/j.1365-2443.2010.01388.x)

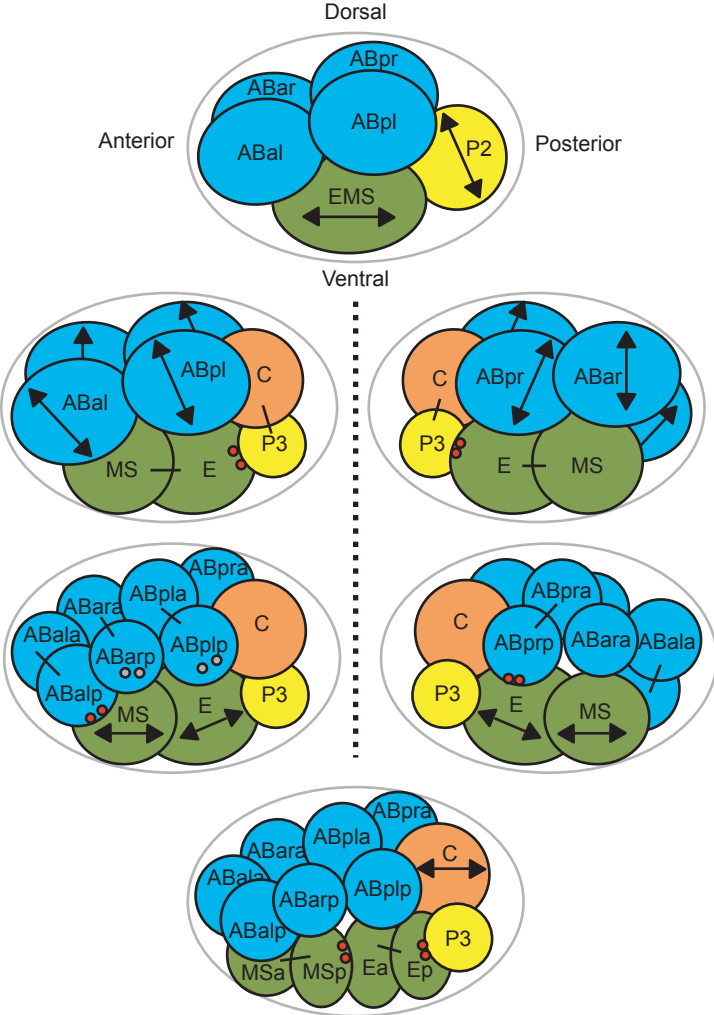

**Figure S1**

Supplement: Supplementary file 1 [file gtc0015-0397-SD1.pdf]

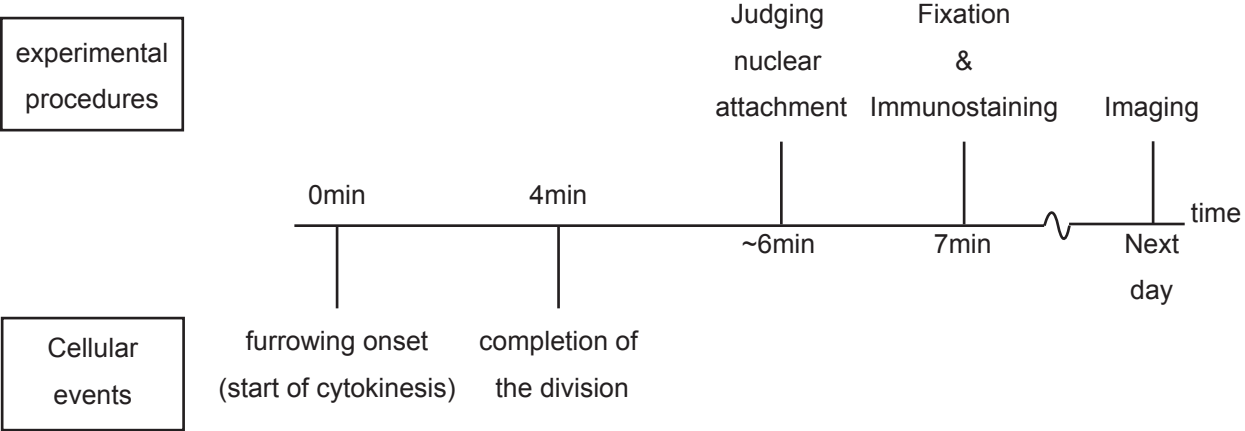

**Figure S2**

Supplement: Supplementary file 2 [file gtc0015-0397-SD2.pdf]
